# Projective Indicators of Underlying Dental Anxiety in Children with Positive Frankl Behavior: A Draw-a-Person Assessment During Caries Removal

**DOI:** 10.3390/children13081008

**Published:** 2026-07-29

**Authors:** Zornitsa Lazarova, Nadezhda Mitova

**Affiliations:** Department of Pediatric Dentistry, Faculty of Dental Medicine, Medical University-Sofia, 1 Georgi Sofiyski St., 1431 Sofia, Bulgaria; z.lazarova@fdm.mu-sofia.bg

**Keywords:** underlying anxiety, Frankl Behavior Rating Scale, Draw-a-Person Test, Brix 3000, pediatric patients

## Abstract

**Highlights:**

**What are the main findings?**

- Children with positive Frankl behavior (scores 3–4) may still exhibit indicators of underlying dental anxiety when assessed using projective drawing methods.
- Chemo-mechanical caries removal with Brix 3000 is associated with higher post-treatment drawing size compared with rotary instrumentation.

**What are the implications of the main findings?**

- Behavioral rating scales such as the Frankl scale assess observable behavior but may not capture internal emotional states in preschool children.
- Minimally invasive chemo-mechanical techniques may improve perceived psychological comfort during pediatric dental treatment.

**Abstract:**

**Background**: Positive behavior during dental treatment does not necessarily indicate the absence of anxiety in children. Behavioral assessment scales, such as the Frankl Behavior Rating Scale, evaluate observable behavior but may fail to identify underlying emotional tension that may accompany dental treatment. **Objectives**: To evaluate indicators of underlying emotional tension in children classified as cooperative based on the Frankl Behavior Rating Scale (scores 3 and 4) undergoing conventional caries removal or chemo-mechanical excavation with Brix 3000. **Methods**: Sixty children aged 4–6 years with positive (score 3) or definitely positive (score 4) Frankl behavior were assigned to two treatment groups: conventional caries removal using rotary instruments (n = 30) and chemo-mechanical excavation with Brix 3000 (n = 30). Projective indicators potentially associated with emotional tension were indirectly assessed using the Draw-a-Person Test. Drawings were obtained at home (baseline), immediately before treatment, and immediately after treatment. Figure height was used as a projective indicator that may reflect emotional tension. **Results**: A marked reduction in figure height was observed immediately before treatment in both groups, consistent with increased emotional tension despite cooperative behavior. Baseline comparison revealed no statistically significant difference between the treatment groups (*p* = 0.842). Following treatment, figure height increased significantly in both groups. Children treated with Brix 3000 demonstrated significantly greater post-treatment figure heights than those treated with rotary instruments (*p* = 0.002), suggesting more favorable projective indicators that may reflect lower emotional tension and improved psychological comfort. **Conclusions**: Cooperative behavior classified by the Frankl Behavior Rating Scale does not exclude the presence of underlying emotional tension that may be associated with anxiety in children undergoing dental treatment. The findings suggest that chemo-mechanical excavation with Brix 3000 may provide greater psychological comfort and potential reduction in indicators consistent with projective indicators of emotional tension than conventional rotary instrumentation. Behavioral assessment alone may not fully capture emotional responses to fully evaluate the emotional state of pediatric dental patients.

## 1. Introduction

Dental anxiety and fear associated with dental treatment constitute major concerns in pediatric dentistry [1,2]. These factors can significantly complicate the provision of dental care and adversely affect children’s future behavior and attitudes toward dental procedures [3].

Data from various studies indicate that between 20% and 30% of children experience fear and anxiety related to dental treatment, with the highest prevalence observed between the ages of 4 and 6 years [3,4,5,6]. At this age, children often have not yet developed adequate coping skills to manage stressful situations and may perceive the dental environment as unfamiliar and potentially threatening [7,8]. Negative emotional experiences during dental treatment may substantially hinder the delivery of dental care. Negative emotional experiences associated with dental treatment may significantly complicate the management of pediatric patients during dental procedures. Over time, these adverse experiences may shape children’s future dental care-seeking behavior, diminish their acceptance of subsequent treatment, and contribute to less favorable oral health outcomes [8,9,10].

The Frankl Behavior Rating Scale is routinely applied in pediatric dentistry to assess children’s behavior during dental treatment [11,12]. However, cooperative behavior during dental treatment does not necessarily reflect the absence of anxiety and fear, as these emotional responses may not be apparent during the clinical examination. Some children may appear cooperative despite experiencing internal emotional tension consistent with anxiety-like states and uncertainty about the anticipated procedure [3,13,14].

Projective methods have been used as complementary approaches for exploring children’s emotional state and may provide additional information that is not evident from behavioral observation alone [3,14]. Among these methods, the Draw-a-Person Test is frequently used, with the size of the drawn figure being regarded as an indirect indicator of emotional tension and fear in some projective assessment frameworks [15,16,17].

Conventional treatment of carious lesions involves the use of rotary instruments and is frequently associated with unpleasant sensory stimuli, including noise, vibrations, pressure, and the anticipation of pain. These factors may contribute to increased emotional tension and anxiety, even among children who appear cooperative during dental treatment [8,9,18].

Contemporary minimally invasive dentistry has increasingly focused on more conservative approaches for managing dental caries in young patients [19,20]. An alternative to conventional cavity preparation is chemo-mechanical excavation using Brix 3000. Brix 3000 is an enzymatic agent based on the proteolytic enzyme papain. It selectively softens irreversibly infected carious dentin, facilitating its removal with a hand excavator. This approach may result in reduced discomfort and improved psychological comfort for children compared with conventional rotary instrumentation [21,22]. However, evidence regarding the impact of different treatment approaches on underlying anxiety in children who exhibit cooperative behavior remains limited.

Despite the widespread use of behavioral rating scales in pediatric dentistry, it remains unclear whether children who exhibit positive behavior during dental treatment experience unrecognized emotional tension that is not detected through standard behavioral assessment. The present study compared chemo-mechanical caries removal using Brix 3000 with conventional rotary caries removal in children aged 4–6 years with positive Frankl behavior. The primary outcome was the change in projective indicators of emotional tension, assessed using the Draw-a-Person Test.

The present study aimed to compare projective indicators of underlying emotional tension in children with positive Frankl behavior (scores 3 and 4) undergoing conventional caries removal or chemo-mechanical excavation with Brix 3000, using a two-way repeated-measures design.

## 2. Materials and Methods

### 2.1. Study Design, Participants and Ethics

This prospective parallel-group controlled clinical study was conducted to compare two caries removal techniques in children with positive Frankl behavior.

The study protocol followed the principles of the Declaration of Helsinki and received approval from the Ethics Committee of the Medical University of Sofia (Protocol No. KENIMUS 05/20, approved on 20 February 2019). Children were recruited and all clinical procedures were performed at the Faculty of Dental Medicine, Medical University of Sofia, Sofia, Bulgaria.

The informed consent forms signed by the parents or legal guardians were those approved by the Ethics Committee under Protocol No. KENIMUS 05/20.

Inclusion criteria:

- Children classified with Frankl scores of 3 or 4, corresponding to positive and definitely positive behavior categories, respectively (i.e., cooperative behavior classification rather than emotional state assessment).
- Presence of at least one carious lesion (occlusal or approximal) classified as International Caries Detection and Assessment System, code 5
- Absence of acute symptoms, such as spontaneous or night pain, at the time of examination and no radiographic or clinical evidence of periapical pathology.
- Written informed consent obtained from a parent or legal guardian prior to participation.

Exclusion criteria:

- Children with Frankl Behavior Rating Scale scores of 1 or 2, corresponding to definitely negative and negative behavior categories, respectively.
- Children presenting acute symptoms at the time of clinical evaluation.
- Absence of parental or legal guardian consent for study participation.

Eligible participants who fulfilled all inclusion criteria and none of the exclusion criteria were assigned to one of the two intervention groups by the treating clinician at the time of treatment. The choice of treatment modality was made according to the treating clinician’s clinical judgment. Because treatment allocation was based on clinical judgment rather than randomization, differences in baseline characteristics not captured by the eligibility criteria cannot be completely excluded.

This was a prospective controlled clinical study without randomization; therefore, no random allocation sequence was generated and no allocation concealment procedures were used. All participants fulfilled identical eligibility criteria prior to group assignment.

All dental procedures, behavioral assessments using the Frankl Behavior Rating Scale, and measurements of the Draw-a-Person drawings were performed by the same experienced pediatric dentist under standardized clinical conditions. No local anesthesia was administered to any participant in either study group during the caries removal procedures. Relative isolation of the operative field was achieved using cotton rolls and saliva suction in all cases.

A total of 78 children were assessed for eligibility. Eighteen children were excluded because they exhibited negative (score 2) or definitely negative (score 1) behavior according to the Frankl Behavior Rating Scale and therefore did not meet the inclusion criteria. The remaining 60 children were enrolled in the study. No formal a priori sample size calculation was performed. The sample size was determined by the number of consecutive eligible children who met the predefined inclusion criteria and whose parents provided written informed consent during the study period. The study population comprised two intervention groups:

- Group 1—30 children treated using conventional caries removal with rotary instruments.
- Group 2—30 children treated using chemo-mechanical excavation with Brix 3000.

**Figure 1:**
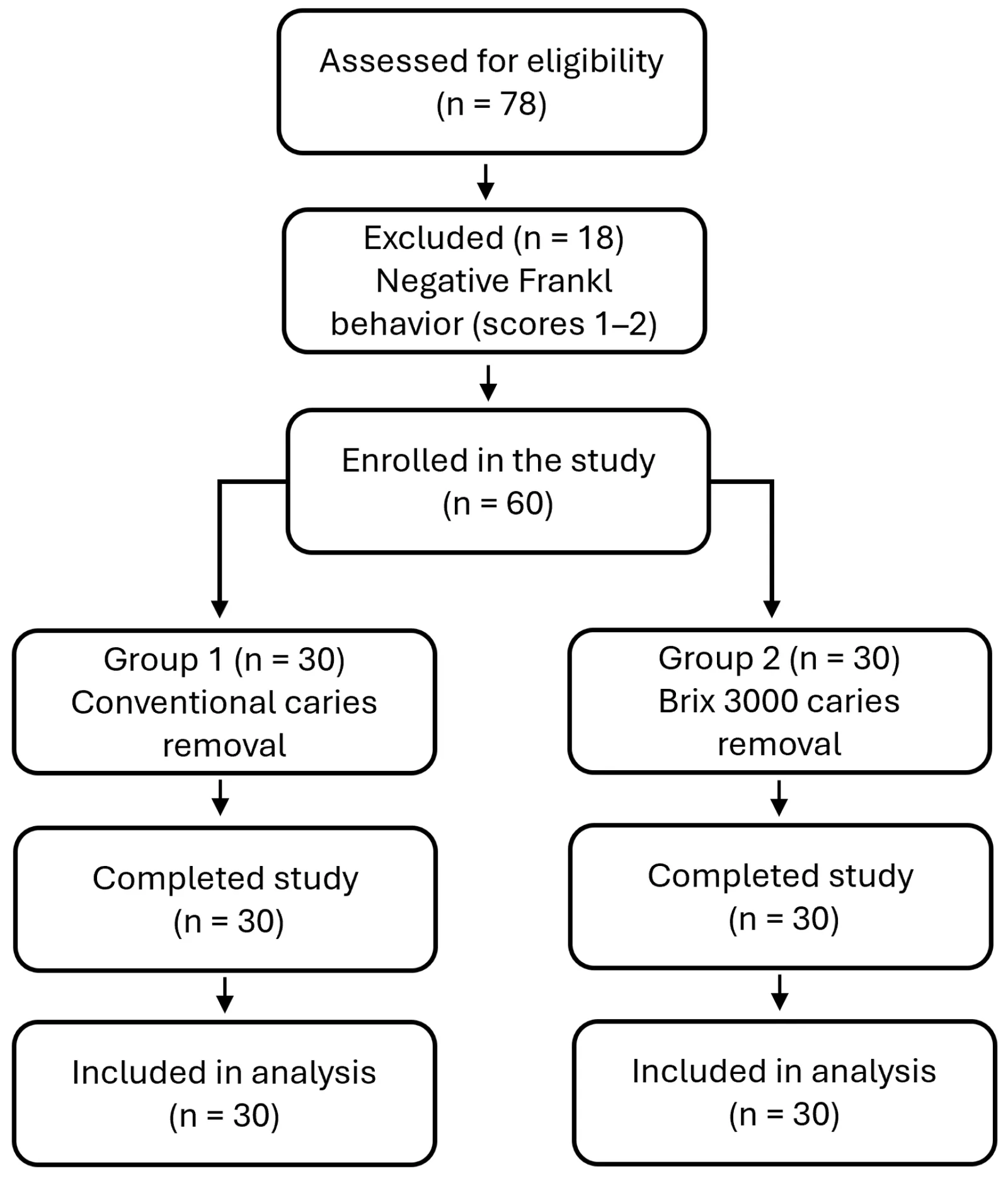
Flowchart of participant selection and allocation throughout the study.

The flow of participant recruitment, eligibility assessment, exclusion, and allocation to the intervention groups is presented in Figure 1.

Blinding was not implemented in this study. Owing to the nature of the interventions, neither the participants nor the treating clinician could be blinded to the treatment received. Behavioral assessments using the Frankl Behavior Rating Scale and measurements of the Draw-a-Person drawings were performed by the same experienced pediatric dentist who delivered the interventions; therefore, outcome assessment was not blinded.

### 2.2. Conventional Cavity Preparation (Group 1)

The procedure involved access to the carious lesion, followed by selective removal of the carious tissue. Carious dentin adjacent to the enamel–dentin junction and cavity walls was removed, while infected dentin from the cavity floor was eliminated and affected dentin with remineralization potential was preserved. A calcium hydroxide liner (Biner LC, Meta Biomed Co., Ltd., Cheongju, Republic of Korea) was subsequently applied, followed by restoration with a compomer material (Glasiosite, VOCO, Cuxhaven, Germany).

### 2.3. Chemo-Mechanical Caries Excavation with Brix 3000 (Group 2)

For chemo-mechanical excavation, Brix 3000 (BRIX Medical Science, Buenos Aires, Argentina) was applied in a small quantity to the carious cavity and left in place for 2 min following the manufacturer’s recommendations. After softening of the infected dentin, the altered tissue was removed with a sickle excavator (Koine, Koine Italia snc, Milan, Italy), whereas the affected dentin was maintained. The restorative procedure was identical to that described for Group 1.

For both intervention groups, the endpoint of caries excavation was determined using fluorescence-assisted caries detection with the Proface^®^ camera (W&H Dentalwerk Bürmoos GmbH, Bürmoos, Austria). Caries excavation followed the principles of selective caries removal described by Bjørndal et al. [23], aiming to preserve affected dentin with remineralization potential. Fluorescence assessment served as an adjunctive tool for identifying the excavation endpoint. Excavation was terminated when pink fluorescence was detected, corresponding to affected dentin with remineralization potential, thereby minimizing unnecessary removal of dental tissue [24].

### 2.4. Method for Behavioral Assessment Using the Frankl Behavior Rating Scale

Children’s cooperative behavior during dental treatment was categorized using the Frankl Behavior Rating Scale, which includes four behavioral levels ranging from definitely negative to definitely positive:

- Definitely Negative (Score 1): Refusal of treatment, forceful crying, fearfulness, or any other overt evidence of extreme negativism.
- Negative (Score 2): Reluctance to accept treatment, uncooperativeness, and some evidence of a negative attitude, although not pronounced (e.g., sullen or withdrawn behavior).
- Positive (Score 3): Acceptance of treatment; at times cautious; willingness to comply with the dentist, occasionally with reservation, but generally follows the dentist’s directions cooperatively.
- Definitely Positive (Score 4): Good rapport with the dentist; interest in dental procedures; laughing and enjoying the situation; verbal communication without signs of fear.

Frankl assessment was performed only before treatment, as the scale was used to select children exhibiting cooperative behavior during the dental procedure. The aim of the study was not to evaluate behavioral changes following treatment, but rather to assess the presence of indirect indicators of underlying anxiety in children who outwardly demonstrated positive behavior in the dental setting.

### 2.5. Method for Assessing Indirect Indicators of Underlying Anxiety Using the Draw-a-Person Test

The Draw-a-Person Test was used to indirectly assess projective indicators of emotional tension that may be associated with anxiety-like responses in children in the context of dental treatment. Each child was asked to draw a person at three different time points: at home, immediately before treatment, and after completion of the treatment. An identical white A4 sheet of paper was provided to all participants.

The first figure drawn at home was considered the baseline reference because the home environment was expected to be less directly associated with treatment-related stress than the dental clinic setting.

The height of the drawn figure was used as a projective drawing parameter reflecting changes in expressive output under different emotional conditions. The drawings were measured in centimetres by determining the distance between the two most distant points of the figure. Each drawing was measured once by the same experienced pediatric dentist using a ruler according to a standardized measurement protocol. Owing to the nature of the study, the examiner was not blinded to treatment allocation because the same clinician performed both the clinical procedures and the measurement of the drawings. Repeated measurements and formal intra- or inter-examiner reliability assessments were not performed. Figure height was selected because it represents an objective and quantifiable drawing characteristic that allows consistent comparison across drawings obtained at different time points and was predefined as the primary outcome measure of the present study. A smaller figure size was considered a potential projective indicator of increased emotional tension. Such changes in expressive output may be associated with anxiety-like responses related to the anticipated or completed dental treatment, whereas a larger figure size was considered indicative of lower emotional tension.

Longitudinal changes in figure height were analyzed using a two-way repeated-measures analysis of variance (ANOVA), with time (home, before treatment, after treatment) as the within-subject factor and treatment modality (Bur vs. Brix 3000) as the between-subject factor. The interaction between time and treatment modality was assessed to determine whether changes in figure height over time differed between the two treatment modalities.

### 2.6. Protocol for Standardized Dental Treatment and Assessment of Underlying Anxiety

The study protocol consisted of the following steps:

- Initial clinical examination and selection of eligible participants.
- Assessment of each child’s behavior before treatment using the Frankl Behavior Rating Scale; only children with positive (score 3) or definitely positive (score 4) behavior were included.
- Instructions were provided to parents regarding the administration of the Draw-a-Person Test at home using a white A4 sheet of paper to obtain a baseline drawing.
- Administration of the Draw-a-Person Test immediately before dental treatment in the clinic waiting area to assess underlying anxiety prior to the dental procedure, regardless of the treatment method to be applied.
- Performance of standardized dental treatment:
  - Group 1: conventional caries removal using rotary instruments;
  - Group 2: chemo-mechanical excavation with Brix 3000.
- Administration of the Draw-a-Person Test immediately after completion of treatment in the clinic waiting area to assess underlying anxiety following the dental procedure.

### 2.7. Statistical Analysis

Statistical analyses were conducted using IBM SPSS Statistics software, version 19.0 (IBM Corp., Armonk, NY, USA).

Continuous variables were expressed as mean values with standard deviation (SD), while categorical variables were reported as absolute numbers and percentages.

The association between Frankl behavior scores and treatment modality was examined using the chi-square (χ^2^) test.

Differences in the mean height of the drawn figure between the two independent groups were assessed using the Independent Samples *t*-test. In cases where the assumption of equal variances was not met, the results from the “Equal variances not assumed” row were considered.

The primary outcome variable was the height of the Draw-a-Person figure measured at three time points (home, before treatment, after treatment).

A two-way repeated measures ANOVA was used to analyze changes in figure height across three time points (home, before treatment, after treatment), with time as the within-subject factor and treatment group as the between-subject factor. This approach was chosen to account for within-subject correlations across repeated measurements and to evaluate both time effects and treatment-related differences simultaneously. Mauchly’s test of sphericity was applied, and when violated, Greenhouse–Geisser correction was used. Post hoc pairwise comparisons with Bonferroni adjustment were performed. Effect sizes were reported using partial eta squared (η^2^p). Effect sizes were interpreted according to conventional benchmarks for η^2^p, with values of approximately 0.01, 0.06, and 0.14 indicating small, medium, and large effects, respectively.

A *p*-value < 0.05 was considered statistically significant.

## 3. Results

Table 1 presents the distribution of the studied children according to their Frankl behavior rating (scores 3 and 4) and the treatment method used.

As shown in Table 1, 32 children were classified as score 3 and 28 children as score 4 according to the Frankl Behavior Rating Scale. No statistically significant difference was found between the study groups with respect to Frankl behavior scores and treatment method, indicating that the groups were comparable at baseline.

Following confirmation of baseline comparability between the groups, further analyses compared conventional caries removal using rotary instruments with chemo-mechanical caries removal using Brix 3000. The repeated-measures analysis was performed including all participants, without adjustment for baseline covariates.

Table 2 presents the mean height of the figure drawn at home according to the treatment method.

Table 2 shows that the mean height of the figure drawn at home was 25.36 ± 1.09 cm in children treated using rotary instruments and 24.54 ± 2.14 cm in children treated with Brix 3000. The observed difference in baseline figure height between the groups did not reach statistical significance (t = 1.866, *p* = 0.069), suggesting comparable baseline drawing characteristics.

Table 3 presents the mean height of the figure drawn immediately before treatment in the dental clinic, regardless of the treatment method.

Table 3 shows a marked decrease in the height of the drawn figure in both groups, reaching approximately 10 cm.

A two-way repeated measures ANOVA revealed a significant main effect of time (F = 369.46, *p* < 0.001, η^2^p = 0.864), indicating substantial changes in figure height across the three time points. A significant time × treatment interaction was observed (F = 11.66, *p* < 0.001, η^2^p = 0.167), demonstrating that the pattern of change over time differed between the Bur and Brix 3000 groups.

Table 4 presents the mean height of the figure drawn immediately after treatment in the dental clinic, regardless of the treatment method.

As presented in Table 4, figure height increased after the dental procedure in both groups. In the group treated conventionally using rotary instruments, the mean figure height was approximately 16 cm, whereas in the group treated with Brix 3000 it was close to 20 cm. The difference between the groups reached statistical significance (*p* = 0.002).

Pairwise comparisons with Bonferroni correction revealed significant differences between all time points (home vs. before, before vs. after, and home vs. after; all *p* < 0.001), confirming a consistent temporal pattern of change in both groups.

The repeated measures analysis demonstrated significant temporal changes in figure height and a significant time-by-group interaction, indicating that the trajectory of changes differed between the two intervention approaches.

**Figure 2:**
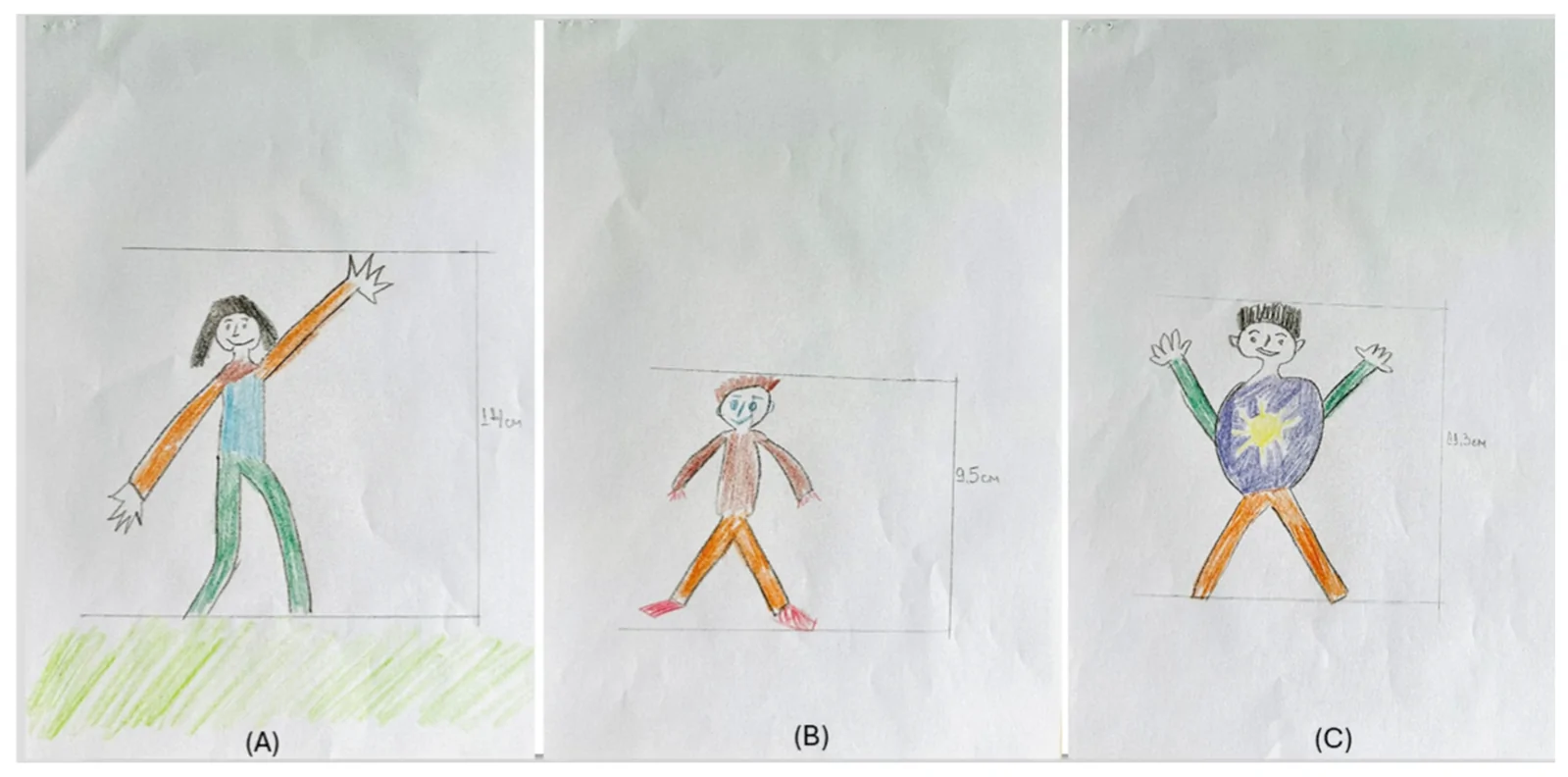
Representative Draw-a-Person Test drawings from a child treated using conventional rotary instrumentation. (**A**) Baseline drawing completed at home. (**B**) Drawing completed immediately before dental treatment. (**C**) Drawing completed immediately after treatment.

Examples of the Draw-a-Person Test drawings obtained at the three assessment time points are presented in Figure 2.

Figure 2 illustrates a representative example of Draw-a-Person Test drawings obtained from a child treated using conventional caries removal with rotary instruments. A marked reduction in figure height is observed immediately before treatment (9.5 cm) compared with the baseline drawing completed at home (14 cm), suggesting increased emotional tension associated with the anticipated dental procedure. Following treatment, the figure height increased to 12.5 cm, indicating reduced projective indicators of emotional tension and partial restoration of psychological comfort. However, the post-treatment figure remained smaller than the baseline drawing, which may suggest the persistence of residual emotional tension following conventional treatment.

**Figure 3:**
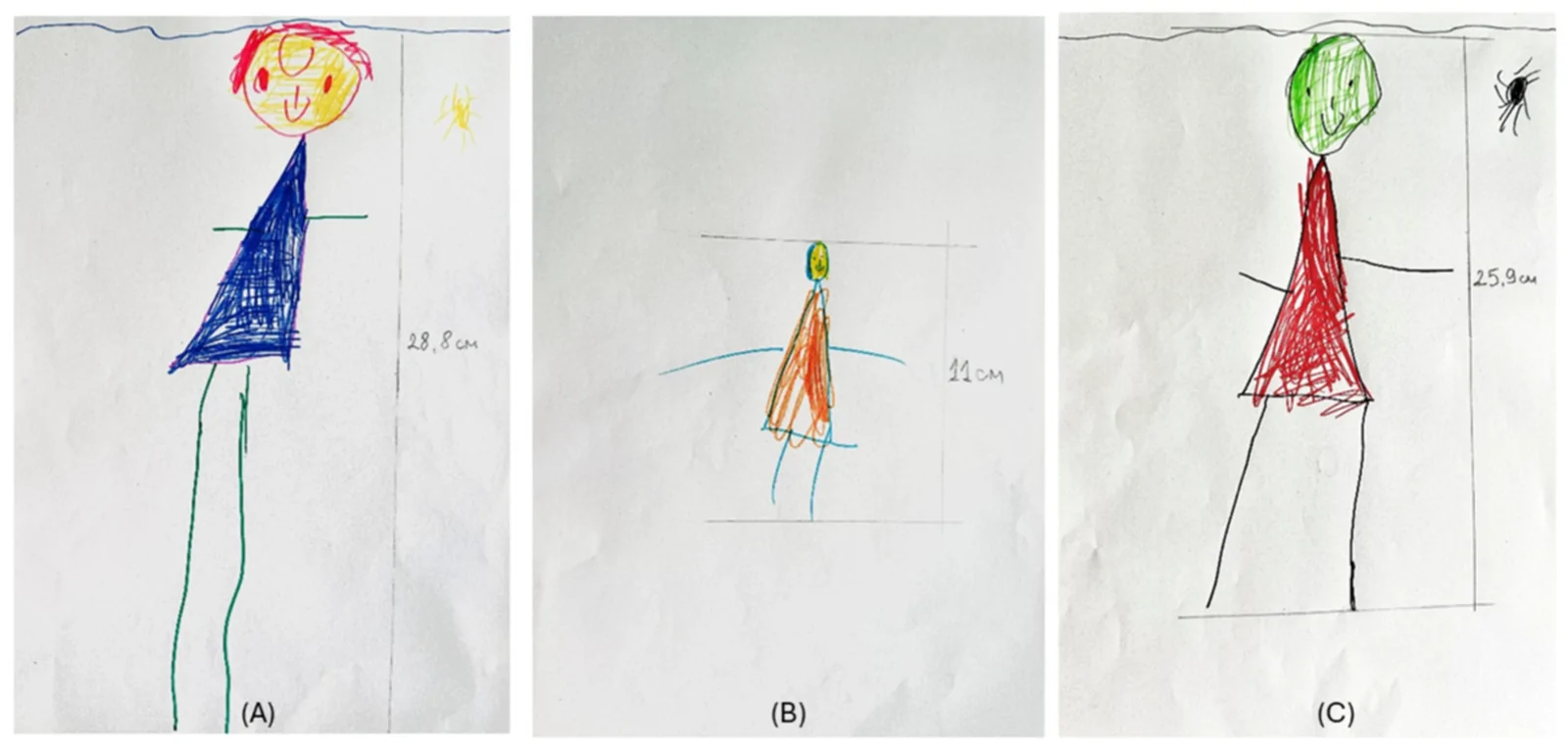
Representative Draw-a-Person Test drawings from a child treated using chemo-mechanical caries removal with Brix 3000. (**A**) Baseline drawing completed at home. (**B**) Drawing completed immediately before dental treatment. (**C**) Drawing completed immediately after treatment.

Figure 3 presents a representative set of Draw-a-Person Test drawings from a participant who underwent minimally invasive caries removal with Brix 3000. A marked reduction in figure height was observed immediately before treatment (11 cm) compared with the baseline drawing completed at home (28.8 cm), suggesting increased emotional tension associated with the anticipated dental procedure. Following treatment, the figure height increased substantially to 25.9 cm, approaching the baseline value. This finding suggests a marked reduction in projective indicators of emotional tension and restoration of psychological comfort after treatment with Brix 3000.

The representative drawings shown in Figure 2 and Figure 3 visually support the findings of the study, demonstrating increased emotional tension before treatment and reduced projective indicators of emotional tension following treatment, particularly in children treated with Brix 3000.

## 4. Discussion

The findings obtained in this study suggest that children classified with favorable Frankl scores may still experience internal emotional responses that are not evident during clinical observation. Although all children included in the study exhibited positive (score 3) or definitely positive (score 4) behavior, a marked reduction in the height of the drawn figures was observed immediately before treatment. This finding suggests increased emotional tension that is not overtly expressed during the clinical examination and may not be fully captured by behavioral assessment alone.

Although the Frankl scale is frequently used for assessing children’s cooperation in dental settings, it mainly reflects externally observed behavioral responses during the clinical procedure. This represents one of its main limitations, as some children may demonstrate cooperative and positive behavior despite experiencing emotional tension, fear, and anxiety. Consequently, behavioral assessment may not always provide a comprehensive reflection of the child’s actual emotional state [25].

The substantial reduction in figure height immediately before treatment suggests increased emotional tension associated with anticipation of the forthcoming dental procedure. The mean height of the figures drawn immediately before treatment was approximately 10 cm in both groups, regardless of the treatment method used (rotary instrumentation or Brix 3000). The pre-treatment values were comparable between the two groups (*p* = 0.842). These findings suggest similar projective indicators that may reflect emotional tension before treatment, irrespective of the treatment method. This may be explained by the perception of the dental clinic as a stressful environment, particularly among children aged 4–6 years, who may not yet have developed sufficient coping skills to manage stressful situations.

The absence of significant differences between the treatment groups before treatment further suggests that anticipatory emotional responses were similar regardless of the planned caries removal technique.

Following completion of treatment, an increase in the height of the drawn figures was observed in both groups, suggesting changes in projective indicators that may be associated with reduced emotional tension. The mean figure height after treatment was 16 cm in children treated using rotary instruments and approximately 20 cm in children treated with Brix 3000 and the post-treatment figure height differed significantly between the two groups (*p* = 0.002). These findings suggest that chemo-mechanical caries excavation may be associated with greater psychological comfort and more favorable projective indicators that may reflect reduced residual emotional tension following treatment. These findings are further supported by the significant time × treatment interaction observed in the repeated measures ANOVA, indicating that the pattern of changes in figure height over time differed according to the treatment modality.

Although drawing height is not a validated psychometric scale, the within-subject repeated-measures design reduces inter-individual variability in drawing ability. Figure height was not interpreted as a diagnostic measure of anxiety but as a projective indicator of emotional tension examined longitudinally within the same child. Therefore, the findings should be interpreted as evidence of changes in emotional expression rather than as direct measurements of anxiety severity.

This finding may be related to the characteristics of the chemo-mechanical technique itself. Unlike conventional caries removal using rotary instruments, treatment with Brix 3000 eliminates several unpleasant stimuli commonly associated with dental treatment, including noise, vibrations, pressure sensations, and fear of pain. The reduction of these stimuli may contribute to lower emotional stress during treatment and a more rapid restoration of emotional comfort following the procedure [26].

The present findings have important clinical implications, as they indicate that underlying emotional tension, potentially associated with anxiety, may be present even in children classified as cooperative based on the Frankl criteria and may not be identified through behavioral assessment alone. These results highlight the importance of an individualized psychological approach to pediatric patients and emphasize the potential value of tissue-preserving, child-friendly treatment approaches that may reduce emotional tension and improve the overall dental experience for children.

Similar observations were described by Mathur J. et al., who investigated the relationship between children’s drawings and behavior assessed using the Frankl Behavior Rating Scale. Their study included 178 children aged 3–14 years and employed free drawings together with the assessment of so-called stress markers. Their findings suggested that children’s drawings could provide complementary information regarding emotional responses that may remain undetected during routine behavioral assessment [27].

Similar findings were reported by Taravati S., who examined the relationship between anxiety and cooperativeness in 169 children aged 4–12 years. Both the Venham Anxiety Scale and the Frankl Behavior Rating Scale were used in the study. The authors reported that anxiety and cooperativeness, assessed using the Venham and Frankl scales, were closely related; however, the findings also indicated that cooperative behavior may not necessarily exclude the presence of dental anxiety [28].

Guner et al. also explored the potential of children’s drawings as a complementary approach for evaluating emotional responses during pediatric dental care. The authors reported an inverse association between drawing-based parameters and Frankl behavior ratings, suggesting that observable cooperation may not always reflect the child’s internal emotional state during dental treatment [15]. These observations support the potential value of projective drawing characteristics as an adjunct to conventional behavioral assessment.

Evidence supporting the improved psychological comfort associated with minimally invasive treatment approaches has also been reported by Hambire et al. In a randomized clinical trial involving 500 children, the authors demonstrated that the use of art therapy and projective techniques resulted in a significant reduction in dental anxiety and improved cooperativeness as assessed by the Frankl Behavior Rating Scale. They emphasized the importance of psychologically supportive approaches in pediatric dentistry [29]. These findings further support the results of the present study, highlighting the importance of identifying children who may experience underlying emotional tension during dental treatment. A child who appears cooperative may still experience significant emotional distress. Therefore, clinicians should avoid assuming emotional comfort solely based on positive Frankl scores and should consider minimally invasive techniques whenever appropriate.

Interpretation of the findings should consider certain methodological constraints, particularly the limited sample size and the restricted age range (4–6 years), which may reduce the generalizability of the results. Additionally, the sample size was based on the consecutive recruitment of eligible participants during the study period rather than on a priori sample size calculation, which may have influenced the statistical power of the study.

Although statistically significant differences and a large interaction effect (η^2^p = 0.167) were observed, future studies with larger samples and predefined sample size calculations are needed to confirm the present findings.

In addition, because participants were allocated to treatment groups according to the treating clinician’s clinical judgment rather than by randomization, selection bias cannot be completely excluded. Although the baseline Frankl behavior scores and baseline drawing heights did not differ significantly between the groups, unmeasured confounding factors may have influenced treatment allocation and should be considered when interpreting the findings.

Another limitation is the use of the Draw-a-Person Test as an indirect method for assessing children’s emotional responses. Figure height was evaluated as the predefined projective parameter because it represents an objective and quantifiable drawing characteristic that allows consistent comparison across drawings obtained at different time points. Nevertheless, figure height should be interpreted as a projective indicator of emotional tension rather than a direct measure of anxiety. Other drawing characteristics, such as line quality, pressure, anatomical details, and omissions, were not assessed and may provide additional information regarding children’s emotional functioning.

Furthermore, potentially relevant factors, including previous dental experience, first dental visit status, socioeconomic background, individual temperament, parental anxiety, sex, and age-related differences, were not assessed or analyzed separately. These variables may have influenced children’s emotional responses independently of the treatment modality and could have partially contributed to the observed findings. Future studies incorporating multivariable analyses and combined psychological, behavioral, and physiological assessment methods are warranted to provide a more comprehensive understanding of children’s emotional responses during dental treatment.

## 5. Conclusions

Cooperative behavior classified as Frankl scores 3 and 4 may coexist with emotional tension potentially related to anxiety-like responses in children aged 4–6 years undergoing dental treatment. The results demonstrated a significant reduction in the height of the drawn figures immediately prior to treatment, regardless of the cooperative behavior exhibited by the children. Following treatment, an increase in figure height was observed in both groups, with higher values recorded in children treated using Brix 3000. This finding suggests improved psychological comfort and more favorable projective indicators that may reflect reduced residual emotional tension following chemo-mechanical caries removal. The results highlight the importance of an individualized psychological approach and the application of minimally invasive treatment methods in pediatric dentistry.

## Figures and Tables

**Table 1:** Distribution of children according to Frankl Behavior Rating Scale scores (3 and 4) and treatment method.

Method of Treatment	Frankl Scale				Total	
	Score 3		Score 4			
	N	%	N	%	N	%
Bur	18	60%	12	40%	30	100%
Brix 3000	14	46.7%	16	53.3%	30	100%
Total	32	53.3%	28	46.7%	60	100%

χ^2^ = 1.071, *p* = 0.301.

**Table 2:** Mean height of the baseline figure drawn at home according to the treatment method.

Method of Treatment	Figure Height at Home		Ind *t*-Test
	N	Mean ± SD	
Bur	30	25.357 ± 1.0881	t = 1.866 *p* = 0.069
Brix 3000	30	24.540 ± 2.1363	
Total	60	24.948 ± 1.7305	

**Table 3:** Mean height of the figure drawn immediately before treatment according to the treatment method.

Method of Treatment	Figure Height Before Treatment		Ind *t*-Test
	N	Mean ± SD	
Bur	30	10.703 ± 1.7799	t = 0.200 *p* = 0.842
Brix 3000	30	10.797 ± 1.8320	
Total	60	10.750 ± 1.7914	

**Table 4:** Mean height of the figure drawn immediately after treatment in the dental clinic, regardless of the treatment method.

Method of Treatment	Figure Height After Treatment		Ind *t*-Test
	N	Mean ± SD	
Bur	30	16.033 ± 5.1952	t = −3.211 *p* = 0.002
Brix 3000	30	19.970 ± 4.2563	
Total	60	18.745 ± 4.7996	

## Data Availability

The data presented in this study are available on request from the corresponding author. The data are not publicly available due to ethical and privacy considerations related to pediatric participants.

## Abbreviations

The following abbreviations are used in this manuscript:

ANOVA	Analysis of Variance
IBM SPSS	IBM Statistical Package for the Social Sciences
SD	Standard Deviation
χ^2^	Chi-square
η^2^p	Partial eta squared
